# The Antigenicity of Epidemic SARS-CoV-2 Variants in the United Kingdom

**DOI:** 10.3389/fimmu.2021.687869

**Published:** 2021-06-17

**Authors:** Jiajing Wu, Li Zhang, Yue Zhang, Haixin Wang, Ruxia Ding, Jianhui Nie, Qianqian Li, Shuo Liu, Yongxin Yu, Xiaoming Yang, Kai Duan, Xiaowang Qu, Youchun Wang, Weijin Huang

**Affiliations:** ^1^ Division of HIV/AIDS and Sex-Transmitted Virus Vaccines, Institute for Biological Product Control, National Institutes for Food and Drug Control (NIFDC) and WHO Collaborating Center for Standardization and Evaluation of Biologicals, Beijing, China; ^2^ Wuhan Institute of Biological Products, Hubei, China; ^3^ National Vaccine & Serum Institute, Beijing, China; ^4^ Department of Pharmaceutical Engineering, College of Life Science and Technology, Dalian University, Dalian, China; ^5^ Graduate School of Peking Union Medical College, Beijing, China; ^6^ Department of Arboviral Vaccine, National Institutes for Food and Drug Control, Beijing, China; ^7^ China National Biotec Group Company Limited, Beijing, China; ^8^ National Engineering Technology Research Center for Combined Vaccines, Wuhan Institute of Biological Products Co. Ltd., Wuhan, China; ^9^ Translational Medicine Institute, First People’s Hospital of Chenzhou, University of South China, Chenzhou, China

**Keywords:** mutation, monoclonal antibody, pseudotyped virus, neutralization, vaccine, B.1.1.7

## Abstract

To determine whether the neutralization activity of monoclonal antibodies, convalescent sera and vaccine-elicited sera was affected by the top five epidemic SARS-CoV-2 variants in the UK, including D614G+L18F+A222V, D614G+A222V, D614G+S477N, VOC-202012/01(B.1.1.7) and D614G+69-70del+N439K, a pseudovirus-neutralization assay was performed to evaluate the relative neutralization titers against the five SARS-CoV-2 variants and 12 single deconvolution mutants based on the variants. In this study, 18 monoclonal antibodies, 10 sera from convalescent COVID-19 patients, 10 inactivated-virus vaccine-elicited sera, 14 mRNA vaccine-elicited sera, nine RBD-immunized mouse sera, four RBD-immunized horse sera, and four spike-encoding DNA-immunized guinea pig sera were tested and analyzed. The N501Y, N439K, and S477N mutations caused immune escape from nine of 18 mAbs. However, the convalescent sera, inactivated virus vaccine-elicited sera, mRNA vaccine-elicited sera, spike DNA-elicited sera, and recombinant RBD protein-elicited sera could still neutralize these variants (within three-fold changes compared to the reference D614G variant). The neutralizing antibody responses to different types of vaccines were different, whereby the response to inactivated-virus vaccine was similar to the convalescent sera.

## Introduction

The pandemic spread of SARS-CoV-2 has severely affected the worldwide economy and healthcare systems. Therapeutic monoclonal antibodies are the most promising treatment, and vaccines are the best hope for prophylaxis. However, an increasing number of SARS-CoV-2 variants have been reported and have rapidly spread to several countries. For example, D614G rapidly became the dominant strain (1), cluster 5 was transmitted between humans and mink (2), the 501Y.V1(VOC-202012/01, B.1.1.7) variant spread rapidly in the United Kingdom (3), the 501Y.V2(VOC-202012/01, B.1.351) variant appeared in South Africa (4), the 501Y.V3(P1, B.1.1.28.1) variant appeared in Brazil (5), and the COH.20G/677H variant appeared in the USA (6). Accordingly, there is great concern that these mutations might affect antigenicity and lead to the failure of therapeutic antibodies and vaccines.

As early as June 2020, our group systematically analyzed spike mutants with a global frequency greater than 0.3%. We found that the D614G mutation increased the infectivity of SARS-CoV-2, but its antigenicity did not change (7). Recently, we demonstrated that the 501Y.V2 variant can escape neutralization by many monoclonal antibodies and compromises the effectiveness of several polyclonal antibodies (8). This study focused on the epidemic strains in the UK, including VOC-202012/01 and the other four high-prevalence variants. We compared the neutralization activity of monoclonal antibodies and sera elicited by different kinds of vaccines that have been approved or are in clinical research, including the inactivated virus vaccine CoronaVac, mRNA vaccine SW0123, as well as spike-encoding DNA or RBD protein, and convalescent sera from COVID-19 patients.

**Figure 1:**
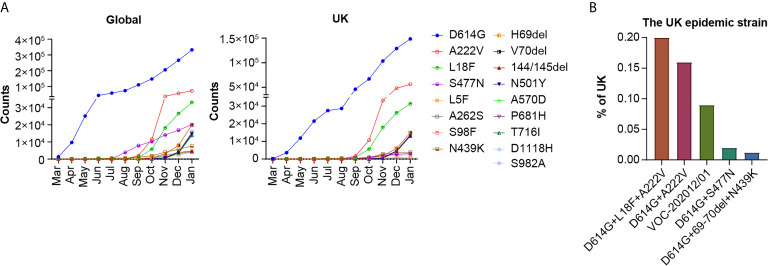
Analysis of mutations and epidemic variants of SARS-CoV-2. **(A)** The numbers of each mutation in the spike protein of SARS-CoV-2 with a frequency above 1% worldwide and in the UK were tracked in GISAID from March 1st 2020 to Jan 13th 2021. The 17 mutations are listed in order of global frequency. **(B)** The percentage of UK epidemic variants on Jan 13th 2021 on GISAID.

Up to January 13, 2021, there were 359, 302 SARS-CoV-2 sequences in the GISAID database, 44% of which were from the UK. We first investigated the growth trend of all mutations with frequencies above 1% globally and in the UK (**Figure 1A**). The most common mutations were D614G, A222V, L18F, and S477N. Since December 2020, the VOC-202012/01 (VOC-202012/01 variant, including multiple mutations 69-70del, 144/145del, N501Y, A570D, P681H, T716I, S982A, and D1118H) has been increasing rapidly. A significant number of genetic changes in the spike protein were speculated to increase infectivity and cause immune escape.

As the recent SARS-CoV-2 variants contain a number of different mutations, we selected the five most frequent natural variants from the UK for further study, which included D614G+L18F+A222V, D614G+A222V, D614G+S477N, D614G+69-70del+N439K, and the VOC-202012/01 strain (**Figure 1B**). We constructed pseudoviruses corresponding to the five variants and 12 single deconvolution mutants of the variants in the D614G genetic background using the VSV vector system. Neutralization activity was compared between the variants and the reference strain D614G.

## Materials and Methods

### Cells

Huh-7 cells were obtained from the Japanese Collection of Research Bioresources (Cat: 0403), and 293T cells were obtained from the American Type Culture Collection (CRL-3216). All the cell lines were cultured in Dulbecco’s modified Eagle’s medium (DMEM, high glucose; HyClone) with 100 U/ml of penicillin–streptomycin solution (GIBCO), 20 mM N-2-hydroxyethylpiperazine-N-2-ethane sulfonic acid (HEPES, GIBCO), and 10% fetal bovine serum (FBS, Pansera ES, PAN-Biotech) at 37°C in a humidified atmosphere comprising 5% CO_2_.

### Monoclonal Antibodies

The monoclonal antibodies cross-binding to the RBD domain, named P2C-1F11, P2B-2F6, 261-262, 151, and 247, which were derived from single B cells from eight individuals infected with SARS-CoV-2 (9), were a kind gift from Professor Linqi Zhang of Tsinghua University. The H014 and H00S022 monoclonal antibodies were obtained from Sino Biological Co., Ltd., which is a technical service company covering many fields of life science research. The two monoclonal antibodies were selected from a phage display antibody library which was generated from RNAs extracted from peripheral lymphocytes of mice immunized with recombinant SARS-CoV RBD. SARS-CoV-2 RBD was used as the target for screening the phage antibody library for potential hits (10). 1F9, 7B8, 4E5, 2F7, 2H10, 10D12, 10F9, 9G11, 11D12, and LK+LH were from Beijing Biocytogen Co., Ltd., which is a new drug research and development company. The preparation method of these Abs was the same as for H014 and H00S022. X593 was developed collaboratively by BeiGene and Singlomics Biopharmaceutical. The underlying mAbs were identified by high-throughput single-cell sequencing of blood samples from recovered patients with COVID-19 at the Advanced Innovation Center for Genomics of Peking University. X593 is being tested in a Phase 2 clinical trial to evaluate its efficacy and safety in patients with mild to moderate COVID-19 (11).

### Convalescent Sera

Sera of convalescent COVID-19 patients were collected from Hubei (CS1-CS5) and Hunan province (CS6-CS10) between March and April 2020. Consent forms were signed prior to blood collection.

### Sera From Vaccinated Participants

The sera elicited by an inactivated virus vaccine (CoronaVac, Sinovac Life Sciences, China) were collected 14 days after the second dose of the standard 0 day and 28 day immunization procedure. Alum adjuvant was used with the inactivated-virus vaccine (12). Ten samples were used in this test. Consent forms were signed prior to blood collection.

The mRNA vaccine (SW0123; Stemirna Therapeutics, Shanghai, China) was administered at a dose of 200 µg on Days 0 and 21, and sera were collected 2 weeks after the second immunization. A total of 14 samples were used for the tests.

### Sera From Immunized Animals

Animals were handled under institutional guidelines for laboratory animal care and use of NIFDC (Beijing, China), and the Animal Care and Use Committee at the NIFDC approved the study protocol.

Mice were immunized with purified SARS-CoV-2 RBD protein with alum adjuvant (20 µg protein) once every 7 days three times subcutaneously. Blood samples were collected 7 days after the third immunization. Serum samples from three mice were pooled for a total of three samples from nine mice.

Guinea pigs were immunized with pcDNA3.1- SARS-CoV-2-Spike plasmid (200 µg per guinea pig) every 14 days three times intramuscularly. The plasmid was diluted to 1 µg/µl with phosphate buffer saline. Four serum samples from four guinea pigs were collected 14 days after the third immunization.

Horses were immunized with the SARS-CoV-2 RBD protein with Freund’s incomplete adjuvant at an initial dose of 3 mg protein *via* the subcutaneous route. After 10 days, 6 mg of RBD protein with Freund’s incomplete adjuvant was injected. The third immunization was performed 10 days after the second immunization with 12 mg of RBD protein with Freund’s incomplete adjuvant. Sera from four horses were collected 7 days after the third immunization.

### SARS-CoV-2 Pseudovirus

The SARS-CoV-2 spike protein expression plasmid pcDNA3.1 was constructed based on the GenBank sequence MN908947, as described previously (7). The replication-defective G*ΔG-VSV (Kerafast, USA) was used as the backbone virus. Cells were transfected with pcDNA3.1-SARS-CoV-2 and simultaneously infected with G*ΔG-VSV, and the supernatant containing the pseudovirus was harvested 24 and 48 h later, aliquoted, and stored at −80°C for further use. Site-directed mutagenesis based on circular PCR and template digestion with *Dpn*I (NEB, USA) was used to construct the mutants of SARS-CoV-2 pseudovirus. The primers used for site-directed mutagenesis are listed in **Supplementary Table 1**. The virus was quantified *via* RT-PCR by detecting the P protein of VSV and diluted with DMEM to 7.0 × 10^4^ TCID_50_/ml as described in our previous paper (13).

### Neutralization Assay

The virus neutralization assay was performed as described in our previous paper (13). The monoclonal antibodies, sera from immunized animals, or convalescent sera were diluted to a certain concentration, followed by a 3-fold serial dilution. The antibodies or sera were mixed with pseudovirus and incubated at 37°C for 1 h. Thereafter, the mixture was added to a 96-well cell culture plate containing 2 × 10^4^ Huh 7 cells in 100 μl per well. The cells were then incubated at 37°C in a humidified atmosphere containing 5% CO_2_. Chemiluminescence signals were detected using the Britelite plus reporter gene assay system (PerkinElmer, USA) after 24 h. The virus neutralization titer was calculated using the Reed–Muench method in PerkinElmer Ensight software. The results were based on three to five repetitions.

### Structure Modeling

The spike protein was modeled based on the Protein Data Bank coordinate set 6VXX, showing the mutation N501Y in S1 and S982A in S2. Pymol program (The PyMOL Molecular Graphics System, Version 2.2.0, Schrödinger, LLC) was used for visualization.

### Statistical Analysis

GraphPad Prism 8 was used for plotting. One-way ANOVA and Holm–Sidak’s multiple comparisons test were used for statistical analysis. The results are shown as means ± SEM. *P <0.05, **P <0.01, ***P <0.005, ****P <0.001.

## Results

### The Neutralization Properties of SARS-CoV-2 Variants Were Affected by Three Mutation in RBD of Spike

**Figure 2:**
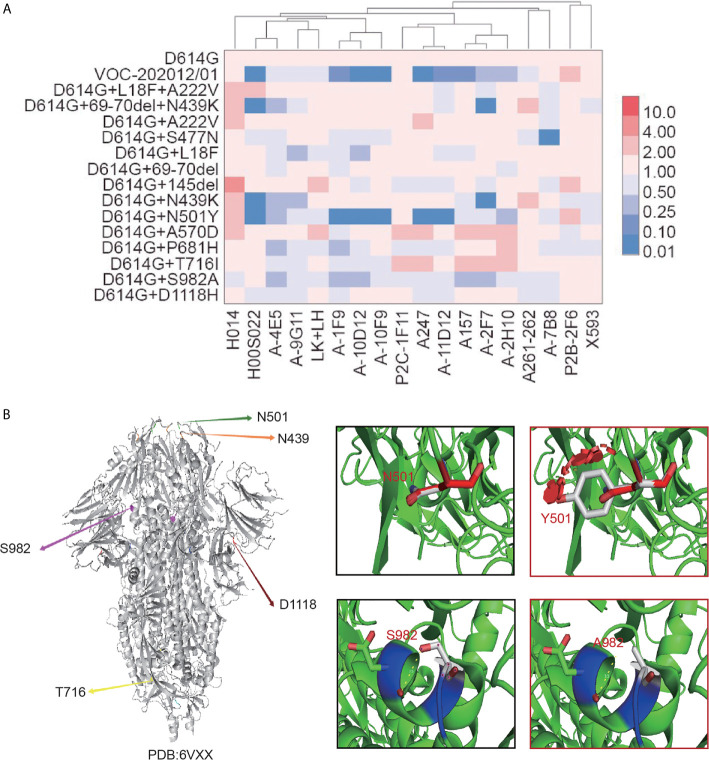
The neutralization activity of mAbs against 16 SARS-CoV-2 variants and mutations. **(A)** Monoclonal antibodies were serially diluted and mixed with equal amounts of the five different SARS-CoV-2 variants and those containing a single mutation. After pre-incubation at 37°C for 1 h, trypsinized Huh 7 cells were added. After cultivation for 24 h, the luminescence of the target cells was measured. The neutralization inhibition rate of the antibody and ID_50_ was calculated using the Reed–Muench method. The data represent the ID_50_ ratio of each variant to that of D614G. The neutralization ability of 19 different monoclonal antibodies (*x axis*) against 16 SARS-CoV-2 variants and mutations (*y axis*) are shown as heatmap. Red represents an increase in neutralization capacity, while blue represents a decrease in neutralization capacity. Four-fold changes were considered statistically significant. **(B)** Structure modeling of the mutation N501Y in S1 and S982A in S2 based on “6VXX”.

To determine whether the existing neutralizing monoclonal antibodies are effective against the five epidemic mutant variants, the neutralizing activity of 18 monoclonal antibodies (mAbs) targeting different areas of the receptor-binding domain was tested (14). Six of the 18 mAbs, including H00S022, 1F9, 10D12, 10F9, A247, and 11D12, displayed significantly reduced neutralizing activity against the VOC-202012/01 variant and variants carrying a single N501Y mutation (**Figure 2A**). Furthermore, mAbs H00S022 and 2F7 lost most of their neutralizing activity against the D614G+69-70del+N439K and N439K+D614G variants. The S477N variant showed decreased susceptibility to mAb 7B8, but most of the other antibodies were still effective (**Figure 2A** and **Supplementary Figure 1**). No decrease of neutralization by any of the mAbs was observed for the variants without mutations in the RBD. The results indicated that the N439K, S477N, and N501Y mutations in the RBD could affect the susceptibility of SARS-CoV-2 variants to neutralization. Structure modeling of the mutation N501Y in S1 and S982A in S2 was showed as **Figure 2B**.

### The Neutralization Response of Convalescent Sera to SARS-CoV-2 Variants Did Not Change Significantly

**Figure 3:**
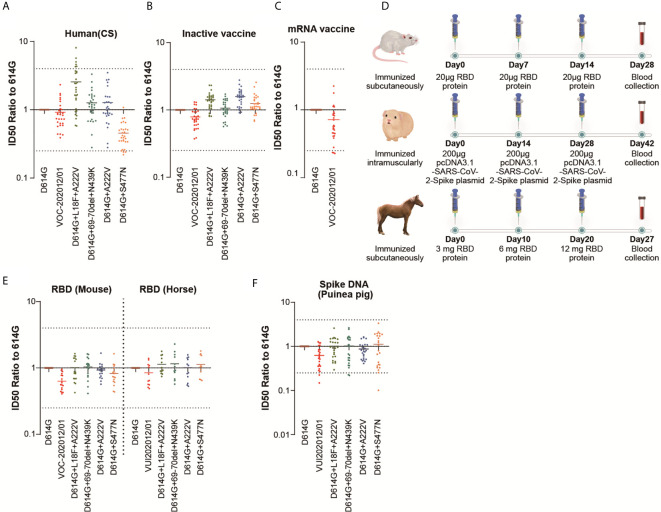
The neutralization activity of polyclonal Abs against the five epidemic variants. Sera were serially diluted and the other procedures were the same as described for **Figure 2A**. Scatter plot of **(A–F)** showing the neutralization ID_50_ ratio of each variant to that of D614G. Each point represents a single result, and the dashed line represents the mean value. The results are a summary of at least three repeated experiments. The dotted line represents 4-fold changes. **(D)** showed the animal immunization scheme.

To investigate whether the five variants may lead to re-infection with SARS-CoV-2, we examined the neutralization ability of sera from convalescent patients who recovered from COVID-19. As shown in **Figure 3A**, the neutralization ability of most of the convalescent sera against VOC-202012/01, D614G+69-70del+N439K and D614G+A222V was not changed (0.9, 1.3 and 1.3-fold compared to D614G, respectively). The convalescent sera showed somewhat increased neutralization activity against D614G+L18F+A222V (2.6-fold) and decreased neutralization activity against D614G+S477N (0.5-fold). Since more than a four-fold change is considered significant in neutralization assays, there was no significant change in the neutralization activity of convalescent sera against these variants.

### The Neutralization Ability of Sera Elicited by Inactivated-Virus Vaccines Against SARS-CoV-2 Variants Did Not Change Significantly

BBIBP-CorV (15) and CoronaVac (12) have been licensed in China. Both vaccines are based on inactivated wild-type virus and have shown good effectiveness in clinical trials (12, 15). To examine whether the protection provided by inactivated-virus vaccines may be compromised by the SARS-CoV-2 variants, the neutralization sensitivity of the variants to vaccine-elicited sera was tested (**Figure 3B**). Our results indicated that the neutralization activity of vaccine-elicited sera against VOC-202012/01 and the other four variants did not change significantly. The sera showed a slightly increased reaction to D614G+L18F+A222V and D614G+A222V, which was similar to the convalescent sera. Moreover, neutralization activity towards D614G+S477N did not decrease.

### The Protective Effect of the mRNA Vaccine (SW0123) Was Not Affected by the VOC-202012/01 Variant

Vaccines based on the experimental mRNA platform have been approved by several countries for the first time due to the urgency of the pandemic. The BNT162b2 vaccine from BioNTech/Pfizer and mRNA-1273 produced by Moderna has been shown to be more than 90% effective in preventing COVID-19 (3, 16). In China, there are also a number of mRNA vaccines in preclinical and clinical stages. Due to limited sample availability, only the VOC-202012/01 variant was tested (**Figure 3C**). The data indicated that VOC-202012/01 did not escape from the mRNA vaccine (SW0123)-elicited sera, where the full-length spike was expressed. This result was in agreement with data on human sera elicited by the BNT162b2 vaccine (3).

### The Neutralization Response of Sera Elicited by Recombinant RBD Protein to VOC-202012/01 Variant Was Slightly Decreased

To predict the effect of the recombinant RBD-based vaccine. Mice and horses were immunized with purified RBD protein from Wuhan-1 strain (**Figure 3D**). The neutralization pattern was slightly different from that of convalescent human sera or inactivated-virus vaccines (**Figure 3E**). However, there was no significant change in the neutralization activity against any of the variants. VOC-202012/01 showed slightly decreased neutralization by sera from both RBD protein-immunized mice and horses, whereas D614G+L18F+A222V and D614G+A222V did not show an increase of neutralization sensitivity.

### The Neutralization Activity of Sera Elicited by a DNA Vaccine to VOC-202012/01 Variant Was Slightly Decreased

Guinea pigs were immunized with the recombinant DNA encoding the full-length spike gene. In our assay, the mutations did not impact neutralization significantly (**Figure 3F**), whereas VOC-202012/01 showed a slight decrease (0.8-fold compared to D614G), which was similar to the RBD-elicited horse sera.

## Discussion

Since December 2020, the SARS-CoV-2 variant named VOC-202012/01, classified as lineage B.1.1.7, has spread rapidly in the United Kingdom. This variant has attracted attention due to several genetic changes in the spike protein, which are speculated to increase infectivity and possibly lead to immune escape. Eight of the 17 mutations in the VOC-202012/01 variant are located in the spike protein, including 69-70del, 144/145del, N501Y, A570D, P681H, T716I, S982A, and D1118H (3). The 69-70del mutation has been described as a dominant mutation in an immune-suppressed individual treated with convalescent plasma, and it is present in several natural variants, including those transmitted in mink (17). The N501Y mutation in the RBD has been identified in a mouse-adapted strain, indicating that it is potentially associated with increased virulence in mice (18). It has also been suggested that the N501Y mutation increases the binding affinity of the spike protein to human ACE2 (19). Furthermore, as the P681H mutation is immediately adjacent to the Furin cleavage site, the proteolytic cleavage during viral maturation is assumed to be influenced (20).

Our study of the neutralization susceptibility of these variants to a panel of mAbs showed that the N501Y mutation of VOC-202012/01 located in the RBD significantly decreased the neutralization activity of seven antibodies. Structure modeling showed that when N501 was mutated into hydrophobic residue Y, it may coordinate with ACE2 better through hydrophobic interaction, improve the interaction conformation of RBD and ACE2, and increase the affinity of RBD and ACE2. In addition, the introduction of a benzene ring formed a large steric effects, which may be one of the reasons for the decrease of neutralization activity of some mAbs caused by N501Y mutation. The single mutation S982A at the S2 fragment of the spike protein also affected the neutralization activity of five mAbs, although not as significantly as N501Y. According to the results of structural analysis, when S982 was mutated to A982, the breaking of hydrogen bonds changed the interaction between this site and surrounding residues, which in turn affected the binding of the mAbs to the S protein, thereby reducing the antibody neutralizing activity. Furthermore, the P681H mutant was also slightly less sensitive to neutralizing mAbs.

However, no evident immune escape of 69-70del was observed in this study, which may be because the selected mAbs were not targeted to the N-terminal domain. Our results therefore indicate that the N501Y, P681H, and S982A mutations might be important antigenic sites of the VOC-202012/01 variant.

L18F and A222V are typical mutations of the B.1.177 lineage. This lineage is also known as 20A.EU1, which is prevalent in England, Denmark and other European countries (21). D614G+L18F+A222V and D614G+A222V were the main epidemic variants before the emergence of VOC-202012/01. Furthermore, the L18F mutation is also present in variants B.1.351and P1, which are reported to exhibit a significant capacity to escape from mAbs and vaccines (22). This study showed that A222V was more sensitive to most mAbs, whereas L18F was slightly resistant to some mAbs. Both the D614G+L18F+A222V and D614G+A222V variants showed increased sensitivity to convalescent sera and sera elicited by inactivated-virus vaccines, which may be caused by the A222V mutation.

Since June 2020, a large number of S477N mutants have appeared and spread rapidly. S477N is the representative mutation site of the B.1.160, B.1.127, and B.1.526 lineages (COVID-19 CoV Genetics Browser, https://covidcg.org/). Lineage B.1.160 grew rapidly from September 2020 in Denmark, Switzerland, France, Britain, and other European countries. In Australia, 60% of the sequences uploaded up to January 2021 contained D614G+S477N. The B.1.526 lineage that recently spread rapidly in New York City, USA, also contains the S477N mutation, which is speculated to significantly impact the epidemic. Since S477N is in the RBD region of S protein, it may slightly change the antigenicity of the virus. Our results showed that S477N escaped from neutralization by the mAb 7B8, which was consistent with the results of another study, which showed broad resistance of this mutant to a group of mAbs (23). Furthermore, S477N was also resistant to neutralization by the human convalescent sera tested in this study, but not to vaccine-elicited sera. The reason for these differences requires further study.

N439K is the representative mutation of lineage B.1.258, in which it always appears together with the 69-70del mutation. This lineage was mainly reported in the UK, Denmark, Germany, Switzerland, Slovenia, and other European countries (24). As it is located in the RBD, the N439K mutation is also resistant to neutralization by some mAbs. However, it did not affect the neutralization activity of polyclonal sera, suggesting that it may not be the key epitope dominant in humans.

Furthermore, we compared the performance of different types of vaccines against these five SARS-CoV-2 variants. Several types of SARS-CoV-2 vaccines are available worldwide, among which the mRNA vaccines BNT162b2 from BioNTech/Pfizer (25) and mRNA-1273 from Moderna (16), as well as the adenoviral vector vaccines Ad26.COV2.S from Johnson & Johnson (26) and ChAdOx1 AstraZeneca (27) are already widely used in western countries. The inactivated-virus vaccines BBIBP-CorV (15) and CoronaVac (12), as well as the adenovirus-based vaccine Ad5-nCoV (28) have been licensed in China. In addition, several recombinant protein vaccines (29) and DNA vaccines (30) are in development. As we were not able to obtain sera from human probands immunized with RBD or DNA vaccines, sera from animals immunized with SARS-CoV-2 RBD or DNA expressing the spike antigen were used to model the immune reactivity of recombinant protein vaccines and DNA vaccines. Mice and guinea pigs were used as common experiment animals. Sera from horses immunized with SARS-CoV-2 RBD protein were used because the RBD-specific equine immunoglobulin F(ab’)2 fragment was also reported as a candidate for the treatment of SARS-CoV-2 (31).

Our results highlight that none of the five variants significantly reduced the neutralization activity of the elicited sera. It also appears that the composition of sera elicited by inactivated-virus vaccines is more similar to convalescent sera, whereas the sera from animals immunized with RBD protein or DNA encoding the full-length spike protein might be different from convalescence sera. The selection of different types of vaccines and the use of RBD or full-length spike as the immunogen requires careful research and comparison. Additional analysis is required to determine the best immunization method and to analyze the mechanism of why different source of spike antigens caused different immune responses.

In summary, this study found that the N501Y, N439K, and S477N mutations significantly decreased the neutralization activity of some monoclonal antibodies. However, they did not significantly affect the neutralization effect of convalescent sera and vaccine-elicited sera. As the epidemic progresses, more complex variants of SARS-CoV-2 could continue to appear. To prevent the failure of therapeutic antibodies and vaccines, it is critical to closely monitor the variants and their antigenicity at all times.

## Data Availability Statement

The raw data supporting the conclusions of this article will be made available by the authors, without undue reservation.

## Ethics Statement

The studies involving human participants were reviewed and approved by the Institutional Review Board of The Center Hospital of Shaoyang. The patients/participants provided their written informed consent to participate in this study. The animal study was reviewed and approved by Animal Care and Use Committee at the NIFDC.

## Author Contributions

YW, LZ, and WH conceived, designed, and supervised the experiments. LZ and YW wrote the manuscript. JW, YZ, RD, HW, QL, SL, and JN performed the experiments. YY, XY, KD, and XQ provided convalescent sera and clinical information. All authors contributed to the article and approved the submitted version.

## Funding

This work was supported by the General Program of the National Natural Science Foundation of China [grant number 82073621], the Bill & Melinda Gates Foundation [Investment ID INV-006379], National Science and Technology Major Projects of Drug Discovery [grant number 2018ZX09101001], and National Science and Technology Major Projects of Infectious Disease [grant number 2017ZX10304402].

## Conflict of Interest

JW, YY and KD were employed by Wuhan Institute of Biological Products Co. Ltd. XY was employed by China National Biotec Group Company Limited.

The remaining authors declare that the research was conducted in the absence of any commercial or financial relationships that could be construed as a potential conflict of interest.

## Supplementary Material

Supplementary Figure 1 The neutralization activity of mAbs against 16 SARS-CoV-2 variants and mutations. Monoclonal antibodies were serially diluted and mixed with equal amounts of the five different SARS-CoV-2 variants and those containing single mutations. After pre-incubation at 37°C for one hour, trypsinized Huh 7 cells were added. After cultivation for 24 h, the luminescence of the target cells was measured. The neutralization inhibition rate of the antibody and ID_50_ (50% inhibitory dilution) were calculated using the Reed-Muench method. The data represent the ID_50_ ratio of each variant compared to D614G. The dotted line shows the four changes. The experiments were repeated three times. Data are shown as the means ± SEM.

Supplementary Table 1 Primers for the construction of the SARS-CoV-2 pseudotyped virus.

Supplementary Table 2 The mutations and epidemic variants of SARS-CoV-2 in the UK.

## References

[1] Plante JA Liu Y Liu J Xia H Johnson BA Lokugamage KG. Spike Mutation D614G Alters SARS-CoV-2 Fitness. Nature (2021) 592(7852):116–21. 10.1038/s41586-020-2895-3 PMC8158177 33106671

[2] Oude Munnink BB Sikkema RS Nieuwenhuijse DF Molenaar RJ Munger E Molenkamp R. Transmission of SARS-CoV-2 on Mink Farms Between Humans and Mink and Back to Humans. Science (2021) 371(6525):172–7. 10.1126/science.abe5901 PMC7857398 33172935

[3] Muik A Wallisch AK Sanger B Swanson KA Muhl J Chen W. Neutralization of SARS-CoV-2 Lineage B.1.1.7 Pseudovirus by BNT162b2 Vaccine-Elicited Human Sera. Science (2021) 371(6534):1152–3. 10.1126/science.abg6105 PMC7971771 33514629

[4] Tegally H Wilkinson E Lessells RJ Giandhari J Pillay S Msomi N. Sixteen Novel Lineages of SARS-CoV-2 in South Africa. Nat Med (2021) 27(3):440–6. 10.1038/s41591-021-01255-3 33531709

[5] Maggi F Novazzi F Genoni A Baj A Spezia PG Focosi D. Imported SARS-CoV-2 Variant P.1 in Traveler Returning From Brazil to Italy. Emerg Infect Dis (2021) 27(4):1249–51. 10.3201/eid2704.210183 PMC8007292 33567246

[6] Tada T Dcosta BM Samanovic-Golden M Herati RS Cornelius A Mulligan MJ. Neutralization of Viruses With European, South African, and United States SARS-CoV-2 Variant Spike Proteins by Convalescent Sera and BNT162b2 mRNA Vaccine-Elicited Antibodies. bioRxiv (2021). 10.1101/2021.02.05.430003

[7] Li Q Wu J Nie J Zhang L Hao H Liu S. The Impact of Mutations in SARS-CoV-2 Spike on Viral Infectivity and Antigenicity. Cell (2020) 182(5):1284–94.e9. 10.1016/j.cell.2020.07.012 32730807 PMC7366990

[8] Li Q Nie J Wu J Zhang L Ding R Wang H. Sars-CoV-2 501y.V2 Variants Lack Higher Infectivity But Do Have Immune Escape. Cell (2021) 184(9):2362–71.e9. 10.1016/j.cell.2021.02.042 PMC7901273 33735608

[9] Ju B Zhang Q Ge J Wang R Sun J Ge X. Human Neutralizing Antibodies Elicited by SARS-CoV-2 Infection. Nature (2020) 584(7819):115–9. 10.1038/s41586-020-2380-z 32454513

[10] Lv Z Deng YQ Ye Q Cao L Sun CY Fan C. Structural Basis for Neutralization of SARS-CoV-2 and SARS-CoV by a Potent Therapeutic Antibody. Science (2020) 369(6510):1505–9. 10.1126/science.abc5881 PMC7402622 32703908

[11] Cao Y Su B Guo X Sun W Deng Y Bao L. Potent Neutralizing Antibodies Against SARS-CoV-2 Identified by High-Throughput Single-Cell Sequencing of Convalescent Patients’ B Cells. Cell (2020) 182(1):73–84 e16. 10.1016/j.cell.2020.05.025 32425270 PMC7231725

[12] Zhang Y Zeng G Pan H Li C Hu Y Chu K. Safety, Tolerability, and Immunogenicity of an Inactivated SARS-CoV-2 Vaccine in Healthy Adults Aged 18-59 Years: A Randomised, Double-Blind, Placebo-Controlled, Phase 1/2 Clinical Trial. Lancet Infect Dis (2021) 21(2):181–92. 10.1016/S1473-3099(20)30843-4 PMC7832443 33217362

[13] Nie J Li Q Wu J Zhao C Hao H Liu H. Quantification of SARS-CoV-2 Neutralizing Antibody by a Pseudotyped Virus-Based Assay. Nat Protoc (2020) 15(11):3699–715. 10.1038/s41596-020-0394-5 32978602

[14] Shi R Shan C Duan X Chen Z Liu P Song J. A Human Neutralizing Antibody Targets the Receptor-Binding Site of SARS-Cov-2. Nature (2020) 584(7819):120–4. 10.1038/s41586-020-2381-y 32454512

[15] Wang H Zhang Y Huang B Deng W Quan Y Wang W. Development of an Inactivated Vaccine Candidate, Bbibp-CorV, With Potent Protection Against SARS-Cov-2. Cell (2020) 182(3):713–21.e9. 10.1016/j.cell.2020.06.008 32778225 PMC7275151

[16] Baden LR El Sahly HM Essink B Kotloff K Frey S Novak R. Efficacy and Safety of the Mrna-1273 SARS-CoV-2 Vaccine. N Engl J Med (2021) 384(5):403–16. 10.1056/NEJMoa2035389 PMC7787219 33378609

[17] Xie X Liu Y Liu J Zhang X Zou J Fontes-Garfias CR. Neutralization of SARS-CoV-2 Spike 69/70 Deletion, E484K and N501Y Variants by BNT162b2 Vaccine-Elicited Sera. Nat Med (2021) 27(4):620–1. 10.1038/s41591-021-01270-4 33558724

[18] Gu H Chen Q Yang G He L Fan H Deng YQ. Adaptation of SARS-CoV-2 in BALB/c Mice for Testing Vaccine Efficacy. Science (2020) 369(6511):1603–7. 10.1126/science.abc4730 PMC7574913 32732280

[19] Starr TN Greaney AJ Hilton SK Ellis D Crawford KHD Dingens AS. Deep Mutational Scanning of SARS-CoV-2 Receptor Binding Domain Reveals Constraints on Folding and ACE2 Binding. Cell (2020) 182(5):1295–1310 e20. 10.1016/j.cell.2020.08.012 32841599 PMC7418704

[20] Zhang L Mann M Syed Z Reynolds HM Tian E Samara NL. Furin Cleavage of the SARS-CoV-2 Spike is Modulated by O-Glycosylation. bioRxiv (2021). 10.1101/2021.02.05.429982 PMC8617502 34732583

[21] Lemey P Ruktanonchai N Hong S Colizza V Poletto C den Broeck FV. SARS-Cov-2 European Resurgence Foretold: Interplay of Introductions and Persistence by Leveraging Genomic and Mobility Data. Res Sq (2021). 10.21203/rs.3.rs-208849/v1

[22] Garcia-Beltran WF Lam EC St Denis K Nitido AD Garcia ZH Hauser BM. Multiple SARS-CoV-2 Variants Escape Neutralization by Vaccine-Induced Humoral Immunity. Cell (2021) 184(9):2523. 10.1016/j.cell.2021.03.013 33930298 PMC8082941

[23] Liu Z VanBlargan LA Bloyet LM Rothlauf PW Chen RE Stumpf S. Identification of SARS-CoV-2 Spike Mutations That Attenuate Monoclonal and Serum Antibody Neutralization. Cell Host Microbe (2021) 29(3):477–488 e4. 10.1016/j.chom.2021.01.014 33535027 PMC7839837

[24] Thomson EC Rosen LE Shepherd JG Spreafico R da Silva Filipe A Wojcechowskyj JA. Circulating SARS-CoV-2 Spike N439K Variants Maintain Fitness While Evading Antibody-Mediated Immunity. Cell (2021) 184(5):1171–1187 e20. 10.1016/j.cell.2021.01.037 33621484 PMC7843029

[25] Polack FP Thomas SJ Kitchin N Absalon J Gurtman A Lockhart S. Safety and Efficacy of the BNT162b2 Mrna Covid-19 Vaccine. N Engl J Med (2020) 383(27):2603–15. 10.1056/NEJMoa2034577 PMC7745181 33301246

[26] Sadoff J Le Gars M Shukarev G Heerwegh D Truyers C de Groot AM. Interim Results of a Phase 1-2a Trial of Ad26.COV2.S Covid-19 Vaccine. N Engl J Med (2021) 384(19):1824–35. 10.1056/NEJMoa2034201 PMC7821985 33440088

[27] Folegatti PM Ewer KJ Aley PK Angus B Becker S Belij-Rammerstorfer S. Safety and Immunogenicity of the ChAdOx1 nCoV-19 Vaccine Against SARS-CoV-2: A Preliminary Report of a Phase 1/2, Single-Blind, Randomised Controlled Trial. Lancet (2020) 396(10249):467–78. 10.1016/S0140-6736(20)31604-4 PMC7445431 32702298

[28] Wu S Zhong G Zhang J Shuai L Zhang Z Wen Z. A Single Dose of an Adenovirus-Vectored Vaccine Provides Protection Against SARS-CoV-2 Challenge. Nat Commun (2020) 11(1):4081. 10.1038/s41467-020-17972-1 32796842 PMC7427994

[29] Yang S Li Y Dai L Wang J He P Li C. Safety and Immunogenicity of a Recombinant Tandem-Repeat Dimeric RBD-Based Protein Subunit Vaccine (ZF2001) Against COVID-19 in Adults: Two Randomised, Double-Blind, Placebo-Controlled, Phase 1 and 2 Trials. Lancet Infect Dis (2021). 10.1016/S1473-3099(21)00127-4 PMC7990482 33773111

[30] Yu J Tostanoski LH Peter L Mercado NB McMahan K Mahrokhian SH. DNA Vaccine Protection Against SARS-CoV-2 in Rhesus Macaques. Science (2020) 369(6505):806–11. 10.1126/science.abc6284 PMC7243363 32434945

[31] Pan X Zhou P Fan T Wu Y Zhang J Shi X. Immunoglobulin Fragment F(Ab’)2 Against RBD Potently Neutralizes SARS-Cov-2 *in vitro* . Antiviral Res (2020) 182:104868. 10.1016/j.antiviral.2020.104868 32659292 PMC7351055

