# Supplementary figures and images for: The Antigenicity of Epidemic SARS-CoV-2 Variants in the United Kingdom
Source: Front Immunol. 2021 Jun 17;12:687869. doi: 10.3389/fimmu.2021.687869 (PMC8247764; doi:10.3389/fimmu.2021.687869)

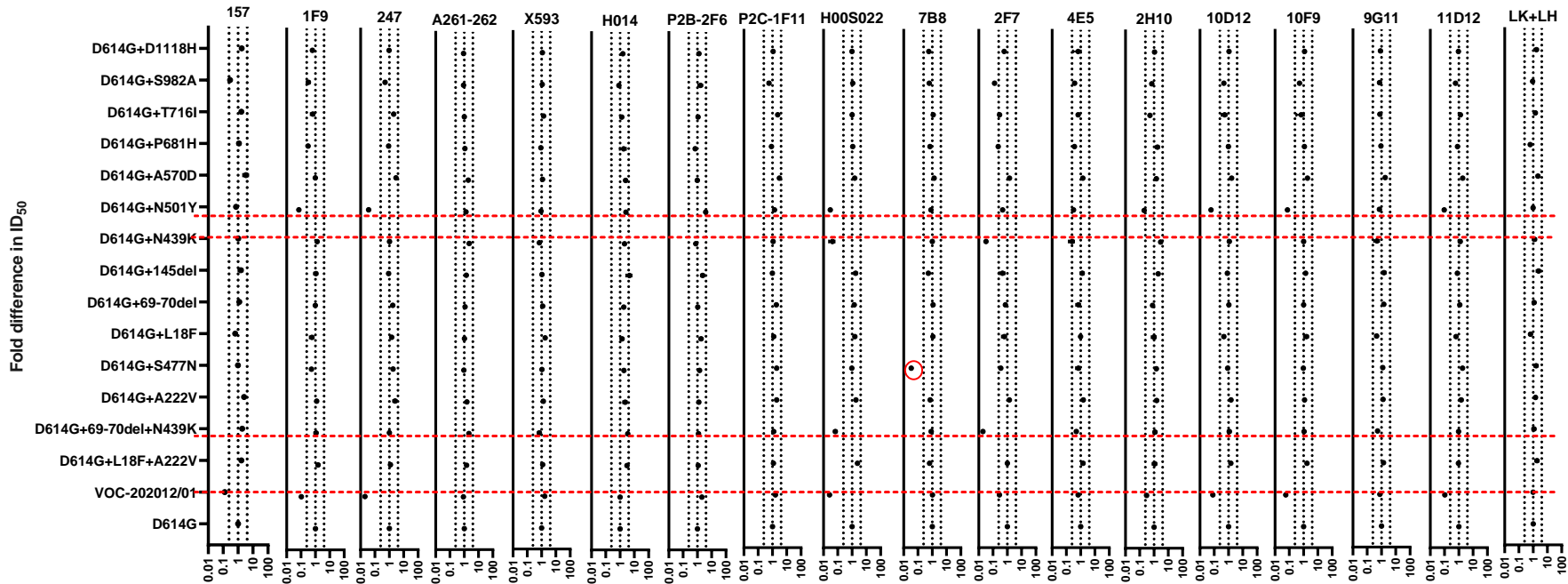

Supplement: Supplementary Figure 1 — The neutralization activity of mAbs against 16 SARS-CoV-2 variants and mutations. Monoclonal antibodies were serially diluted and mixed with equal amounts of the five different SARS-CoV-2 variants and those containing single mutations. After pre-incubation at 37°C for one hour, trypsinized Huh 7 cells were added. After cultivation for 24 h, the luminescence of the target cells was measured. The neutralization inhibition rate of the antibody and ID50 (50% inhibitory dilution) were calculated using the Reed-Muench method. The data represent the ID50 ratio of each variant compared to D614G. The dotted line shows the four changes. The experiments were repeated three times. Data are shown as the means ± SEM. [file Image_1.pdf]
