# Supplementary material for: The Antigenicity of Epidemic SARS-CoV-2 Variants in the United Kingdom
Source: Front Immunol. 2021 Jun 17;12:687869. doi: 10.3389/fimmu.2021.687869 (PMC8247764; doi:10.3389/fimmu.2021.687869)
Supplement: Supplementary Table 1 — Primers for the construction of the SARS-CoV-2 pseudotyped virus. [file Table_1.pdf]

Site	Mutatant	Forward Primer
18	L18F	GGTGAGCAGCCAGTGCGTGAATTTCACCACCAGAACCCAGCTGCCTC
69-70	69-70del	CGTGACCTGGTTCCACGCCATCAGCGGCACCAATGGCACCAAGAGATTC
145	145del	GACCCTTTCCTGGGTGTTTATCATAAGAACAACAAGAGCTGGATGG
222	A222V	ACCTGCCTCAGGGCTTCAGCGTGCTGGAGCCTCTGGTGGACCTG
439	N439K	CTGCGTGATCGCGTGGAACTCTAAGAACCTGGACTCGAAAGTTGGAGGC
477	S477N	GCACCGAGATCTACCAGGCCGGCAACACACCGTGTAATGGCGTGGAGGGC
501	N501Y	AGAGCTACGGCTTCCAGCCTACCTACGGCGTGGGCTACCAGCCTTACAG
570	A570D	CAACAATTCGGCAGAGACATCGACGACACCACAGATGCTGTAAGAGAC
614	D614G	GTGGCCGTGCTGTACCAGGGCGTGAATTGCACCGAGGT
681	P681H	CTACCAGACCCAGACCAATAGCCACAGAAGAGCCAGAAGCGTGGCCAGCC
716	T716I	CAATAATAGCATCGCCATCCCTATCAATTTCACCATCAGCGTGACCAC
982	S982A	TACTCAACGACATCCTGGCGAGACTGGACAAGGTGGAGGCCGA
1118	D1118H	ACGAGCCTCAGATCATCACCACCCACAATACCTTCGTGAGCGGCAA
