# Supplementary material for: The Antigenicity of Epidemic SARS-CoV-2 Variants in the United Kingdom
Source: Front Immunol. 2021 Jun 17;12:687869. doi: 10.3389/fimmu.2021.687869 (PMC8247764; doi:10.3389/fimmu.2021.687869)
Supplement: Supplementary Table 2 — The mutations and epidemic variants of SARS-CoV-2 in the UK. [file Table_2.docx]

|  | Global | | The United Kingdom | |
| --- | --- | --- | --- | --- |
|  | sequences | frequency | sequences | frequency |
| Total | 359,302 | / | 156,705 | / |
| D614G | 332,301 | 92.5% | 148,521 | 94.8% |
| A222V | 71,963 | 20.0% | 56150 | 35.8% |
| L18F | 33,040 | 9.2% | 31,316 | 20.0% |
| S477N | 20,131 | 5.6% | 2,997 | 1.9% |
| H69del | 19,942 | 5.6% | 14,909 | 9.5% |
| V70del | 19,939 | 5.5% | 14,917 | 9.5% |
| N439K | 7,680 | 2.1% | 3,855 | 2.5% |
| N501Y | 14,697 | 4.1% | 13,586 | 8.7% |
| P681H | 15,568 | 4.3% | 13,191 | 8.4% |
| 144/145del | 14,152 | 3.9% | 13,169 | 8.4% |
| T716I | 13,937 | 3.9% | 13,139 | 8.4% |
| L5F | 4,624 | 1.3% | 2,342 | 1.5% |
| A570D | 13,787 | 3.8% | 13,081 | 8.3% |
| D1118H | 13,788 | 3.8% | 13,075 | 8.3% |
| S982A | 13,766 | 3.8% | 12,986 | 8.3% |
| A262S | 4,081 | 1.1% | 3,087 | 2.0% |
| S98F | 4,031 | 1.1% | 862 | 0.6% |
| D614G+L18F+A222V | 33,316 | 9.3% | 30,603 | 19.5% |
| D614G+A222V | 42,281 | 11.8% | 25,614 | 16.3% |
| VOC-202012/01(B.1.1.7) | 14,627 | 4.1% | 13638 | 8.7% |
| D614G+S477N | 20,129 | 5.6% | 3,001 | 1.9% |
| D614G+69-70del+439K | 5,200 | 1.4% | 1,917 | 1.2% |

Supplementary Table 2
